# Deep learning reconstruction for high-resolution computed tomography images of the temporal bone: comparison with hybrid iterative reconstruction

**DOI:** 10.1007/s00234-024-03330-1

**Published:** 2024-03-22

**Authors:** Nana Fujita, Koichiro Yasaka, Sosuke Hatano, Naoya Sakamoto, Ryo Kurokawa, Osamu Abe

**Affiliations:** Department of Radiology, Graduate School of Medicine, The University of Tokyo, 7-3-1 Hongo, Bunkyo-Ku, Tokyo 113-8655 Japan

**Keywords:** Deep learning, Image processing, Temporal bone, Multidetector computed tomography

## Abstract

**Purpose:**

We investigated whether the quality of high-resolution computed tomography (CT) images of the temporal bone improves with deep learning reconstruction (DLR) compared with hybrid iterative reconstruction (HIR).

**Methods:**

This retrospective study enrolled 36 patients (15 men, 21 women; age, 53.9 ± 19.5 years) who had undergone high-resolution CT of the temporal bone. Axial and coronal images were reconstructed using DLR, HIR, and filtered back projection (FBP). In qualitative image analyses, two radiologists independently compared the DLR and HIR images with FBP in terms of depiction of structures, image noise, and overall quality, using a 5-point scale (5 = better than FBP, 1 = poorer than FBP) to evaluate image quality. The other two radiologists placed regions of interest on the tympanic cavity and measured the standard deviation of CT attenuation (i.e., quantitative image noise). Scores from the qualitative and quantitative analyses of the DLR and HIR images were compared using, respectively, the Wilcoxon signed-rank test and the paired *t-*test.

**Results:**

Qualitative and quantitative image noise was significantly reduced in DLR images compared with HIR images (all comparisons, *p* ≤ 0.016). Depiction of the otic capsule, auditory ossicles, and tympanic membrane was significantly improved in DLR images compared with HIR images (both readers, *p* ≤ 0.003). Overall image quality was significantly superior in DLR images compared with HIR images (both readers, *p* < 0.001).

**Conclusion:**

Compared with HIR, DLR provided significantly better-quality high-resolution CT images of the temporal bone.

## Introduction

The temporal bone region is the site of diseases such as cholesteatoma, ossicular malformation, otosclerosis, and trauma. Computed tomography (CT) plays an important role in detecting and diagnosing the extent of those diseases. Ossicle erosion or perforation of the tympanic membrane can be seen in patients with cholesteatoma [1]. Demineralized foci in the otic capsule can be seen in patients with otosclerosis [2]. Because the structures being imaged—the auditory ossicles, tympanic membrane, and so on—are small, high-resolution CT of the temporal bone uses a targeted field of view, thin slices, and dedicated kernels in image reconstruction. Those techniques improve the spatial resolution of the CT images, but they are also associated with increased image noise.

Increasing the tube current can reduce noise in CT images. However, that approach inevitably increases the patient’s radiation exposure. In the 2010s, the iterative reconstruction algorithm gained wide attention for reducing image noise better than conventional filtered back projection (FBP) could. Full iterative reconstruction reduced image noise considerably and improved the quality of CT images [3–8]; however, the associated prolonged image reconstruction time hampered the use of this algorithm in daily clinical practice. An alternative hybrid iterative reconstruction (HIR) algorithm that provides some reduction in image noise within a clinically feasible reconstruction time was subsequently widely accepted [9–11].

Deep learning applications have recently been gaining wide attention in the field of radiology [12–14]. Studies have shown that deep learning can be applied not only to support radiologists in diagnosing diseases [15–17] but also to improve the image reconstruction process. Deep learning reconstruction (DLR) is one such algorithm [18]. In supervised training of DLR, low-quality data and high-quality images are respectively used as the input and reference data. A trained DLR is known to reduce image noise [19], thereby improving the quality of CT images in regions as varied as the head [20, 21], chest [9], abdomen [10, 19, 22, 23], and vertebrae [11, 24, 25]. We therefore hypothesized that DLR can also improve the quality of high-resolution CT of the temporal bone. To our knowledge, no published report has yet assessed how DLR, compared with HIR, might improve the quality of high-resolution CT images in this region. In the present study, we therefore investigated whether DLR, compared with HIR, can improve the quality of such images.

## Methods

This retrospective study was approved by our Institutional Review Board, which waived the requirement for obtaining written informed consent from patients.

### Patients

A radiologist (radiologist A, with imaging experience of 5 years) searched for all consecutive patients who had undergone high-resolution CT of the temporal bone from May 2023 to July 2023. Of 40 patients who met that criterion, those less than 12 years of age (*n* = 4) were excluded from the final analyses, although their images were used in a training session as will be described later. As a result, the study included 36 patients (15 men, 21 women; age, 53.9 ± 19.5 years).

### CT imaging

All patients were imaged using a single CT machine (Aquilion One: Canon Medical Systems, Otawara, Japan). The temporal bone region was imaged in volume scan mode, using these parameters: tube voltage, 120 kVp; tube current, 200 mA; gantry rotation time, 1000 ms; and data collection diameter, 240 mm. Based on the source data, the high-resolution CT images of the right side of the temporal bone were reconstructed with DLR (Advanced intelligent Clear-IQ Engine [with inner-ear standard]: Canon Medical Systems), HIR (Adaptive Iterative Dose Reduction [with FC80]: Canon Medical Systems), and FBP with FC80. FC80 is a kernel for the evaluation of the temporal bone. All the parameters for reconstruction by DLR, HIR, and FBP were the same: field of view, 80 mm; slice thickness/interval, 0.5 mm/0.5 mm; plane, axial. A multiple planar reconstruction from the axial images was performed, and axial and coronal images (slice thickness/interval, 1 mm/1 mm for both) were reconstructed. The resulting images (axial and coronal images with slice thickness/interval of 1 mm/1 mm for both) were used for the study analyses.

### Qualitative image analyses

Two radiologists (readers 1 and 2, with imaging experience of 9 and 5 years, respectively) used the Image J software (https://imagej.net/ij/) to perform the qualitative image analyses. Before that work commenced, a radiologist (radiologist B, with imaging experience of 13 years) randomized the image sets. The readers were blinded to the patient’s clinical background and the image reconstruction algorithm. Earlier, to get used to the study scoring system (to be described shortly), the two blinded readers had taken a training session that used the CT images of the four pediatric patients who had been excluded from the final analysis. The readers were presented with images of two reconstruction algorithms (DLR and FBP, or HIR and FBP) using a default window width of 4500 HU and a window level of 250 HU, which could be modified at the reader’s convenience. The readers independently evaluated the image sets using Likert scales, modified from a previous article [26], as follows:

- Depiction of structures (i.e., osseous spiral lamina, cochlear axis, otic capsule, auditory ossicles, tympanic membrane) on a 5-point scale (5 = better than FBP, 4 = slightly better than FBP, 3 = equal to FBP, 2 = slightly poorer than FBP, and 1 = poorer than FBP)
- Noise on a 5-point scale (5 = less noise than FBP, 4 = slightly less noise than FBP, 3 = noise equal to FBP, 2 = slightly more noise than FBP, and 1 = more noise than FBP)
- Overall image quality on a 5-point scale (5 = better than FBP, 4 = slightly better than FBP, 3 = equal to FBP, 2 = slightly poorer than FBP, and 1 = poorer than FBP)

DLR and HIR images were compared with FBP image, because this method would be expected to reliably detect the difference of image quality [26].

### Quantitative image analyses

Under the supervision of radiologist B, radiologist A placed regions of interest with diameter of 1–1.5 mm on homogenous parts of the otic capsule medial to the cochlea (bone) and tympanic cavity (air) in the axial and coronal images. The locations of them were almost the same between patients. The copy-and-paste function of the region of interest was used to ensure that the location and size were identical on the DLR and HIR images. After the regions of interest had been placed, the mean of the CT attenuation for those regions and the standard deviation of the CT attenuation for air (i.e., quantitative image noise) were recorded. The contrast-to-noise ratio (CNR) was calculated using the formula

$$\text{CNR}=\left({\text{CT}}_\text{BONE}-{\text{CT}}_\text{AIR}\right)/\mathrm{Quantitative}\;\mathrm{image}\;\mathrm{noise},$$

where CT_BONE_ and CT_AIR_ are the CT attenuation of bone and air, respectively. These measurements were also performed by radiologist B, and averaged values were used for the main analyses.

**Fig. 1:**
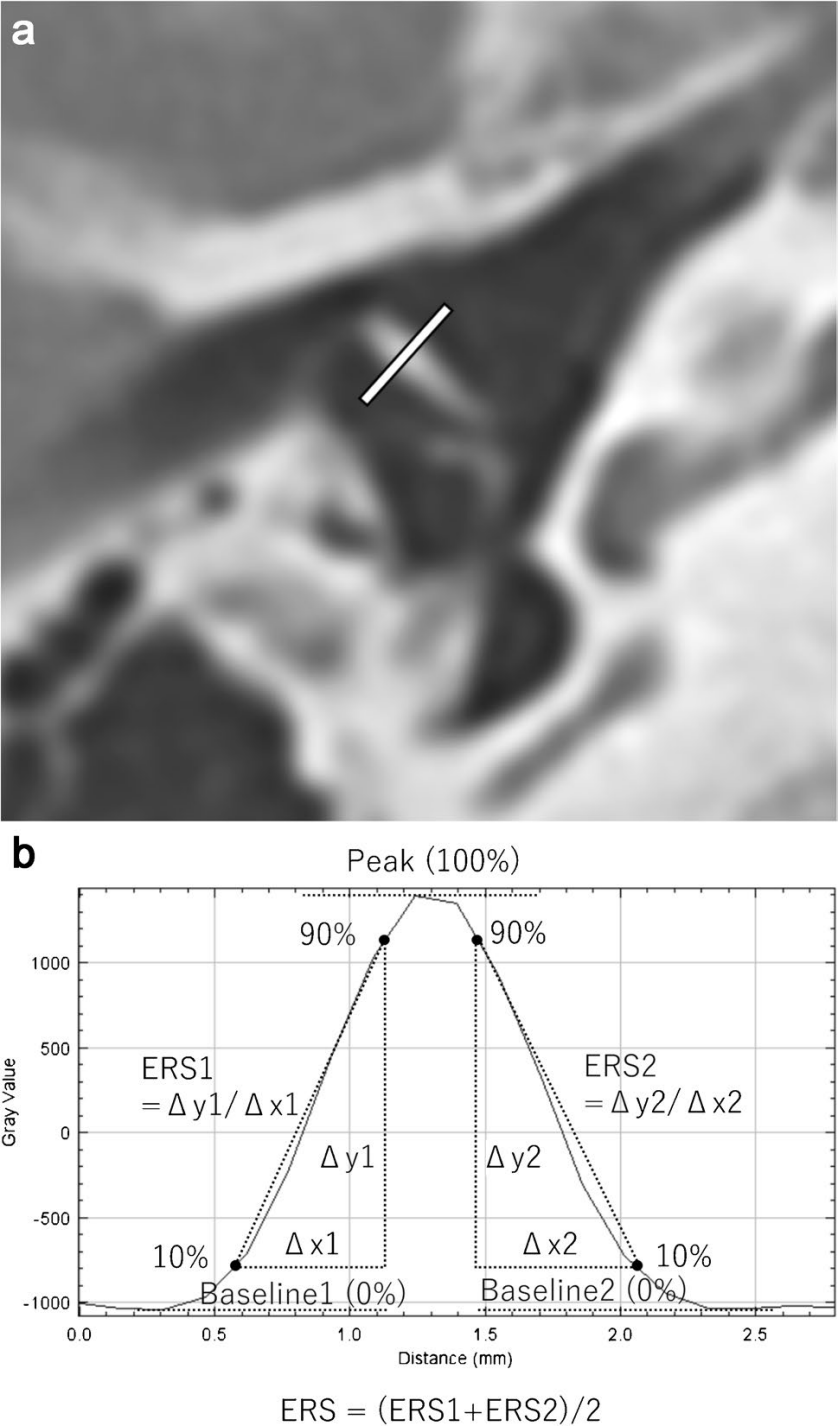
**A** A linear region of interest was placed passing perpendicular to the handle of the malleus on axial image. **b** Based on the CT attenuation profile along the region of interest, the edge rise slope (ERS), which was averaged for both slopes, was calculated

As similar to previous studies [27, 28], radiologist B placed a linear region of interest passing the handle of the malleus on axial image (Fig. 1a). The copy-and-paste function of region of interest was also used in this analysis to ensure that the location and size were identical on the DLR and HIR images. Based on the CT attenuation profile along the region of interest, the edge rise slope [29, 30] was calculated (Fig. 1b).

### Statistical analyses

All statistical analyses were performed using the EZR software application, version 1.55, which is a graphical user interface based on the R software application, version 4.1.2 (The R Foundation for Statistical Computing, Vienna, Austria). Scores from the DLR and HIR qualitative image analyses were compared using the Wilcoxon signed-rank test. Values from the DLR and HIR quantitative image analyses were compared using the paired *t*-test. A *p* value of less than 0.050 was considered to indicate a statistically significant difference. Inter-rater concordance for the quantitative measurements was calculated with intraclass correlation (ICC).

## Results

### Qualitative image analyses

**Fig. 2:**
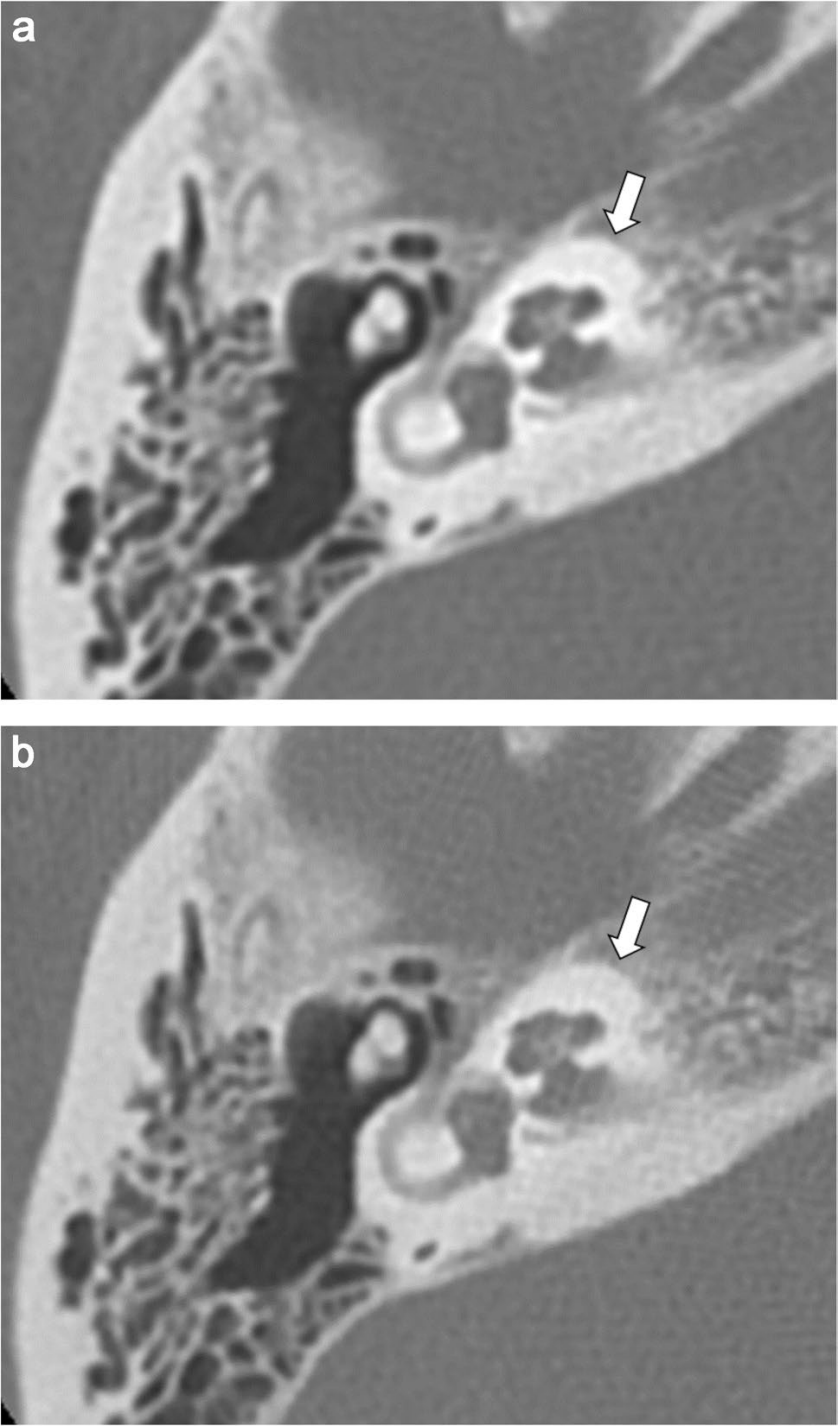
Computed tomography (CT) images of a 70-year-old female patient after **a** deep learning reconstruction (DLR) and **b** hybrid iterative reconstruction (HIR). The depiction of the otic capsule (arrow) was rated as 5/3 and 5/3 (DLR/HIR) by readers 1 and 2, respectively

**Fig. 3:**
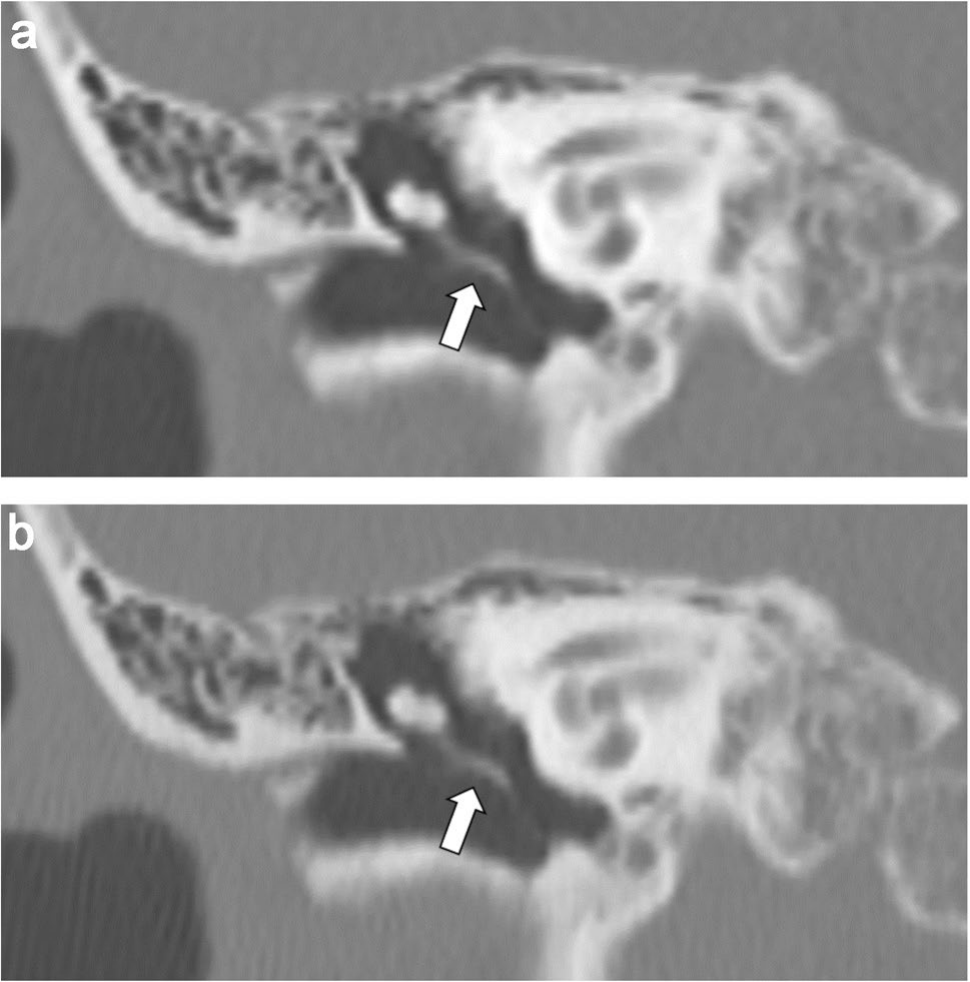
Computed tomography (CT) images of a 73-year-old female patient after **a** deep learning reconstruction (DLR) and **b** hybrid iterative reconstruction (HIR). The depiction of the auditory ossicles (arrow) was rated as 5/3 and 4/3 (DLR/HIR), and the depiction of the tympanic membrane was rated as 5/3 and 3/3 (DLR/HIR) by readers 1 and 2, respectively

**Fig. 4:**
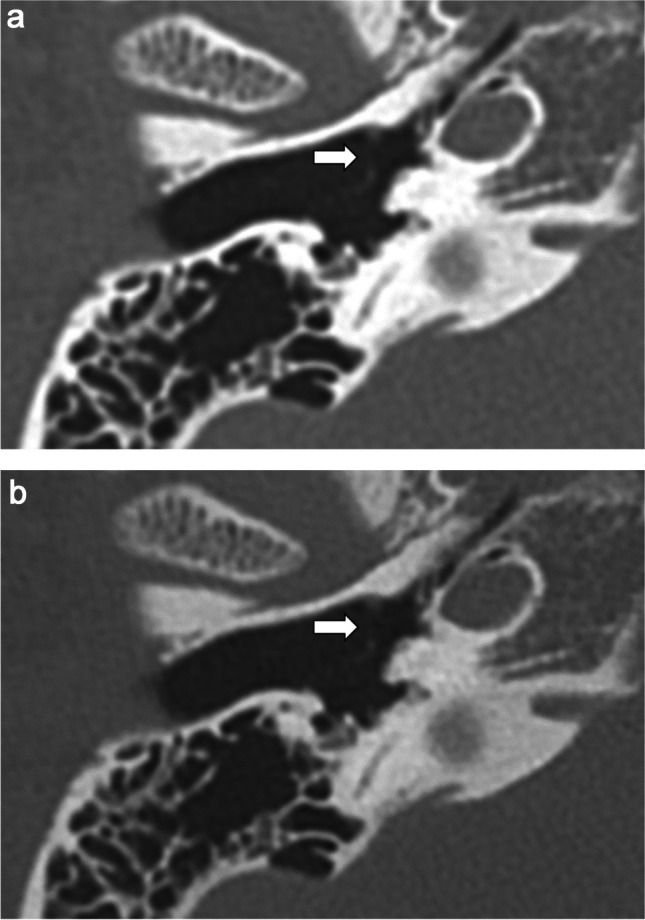
Computed tomography (CT) images of a 58-year-old male patient after **a** deep learning reconstruction (DLR) and **b** hybrid iterative reconstruction (HIR). Perforation of the tympanic membrane (arrow) is more clearly depicted in **a**. The depiction of the tympanic membrane was rated as 5/3 and 4/3 (DLR/HIR) by readers 1 and 2, respectively

**Fig. 5:**
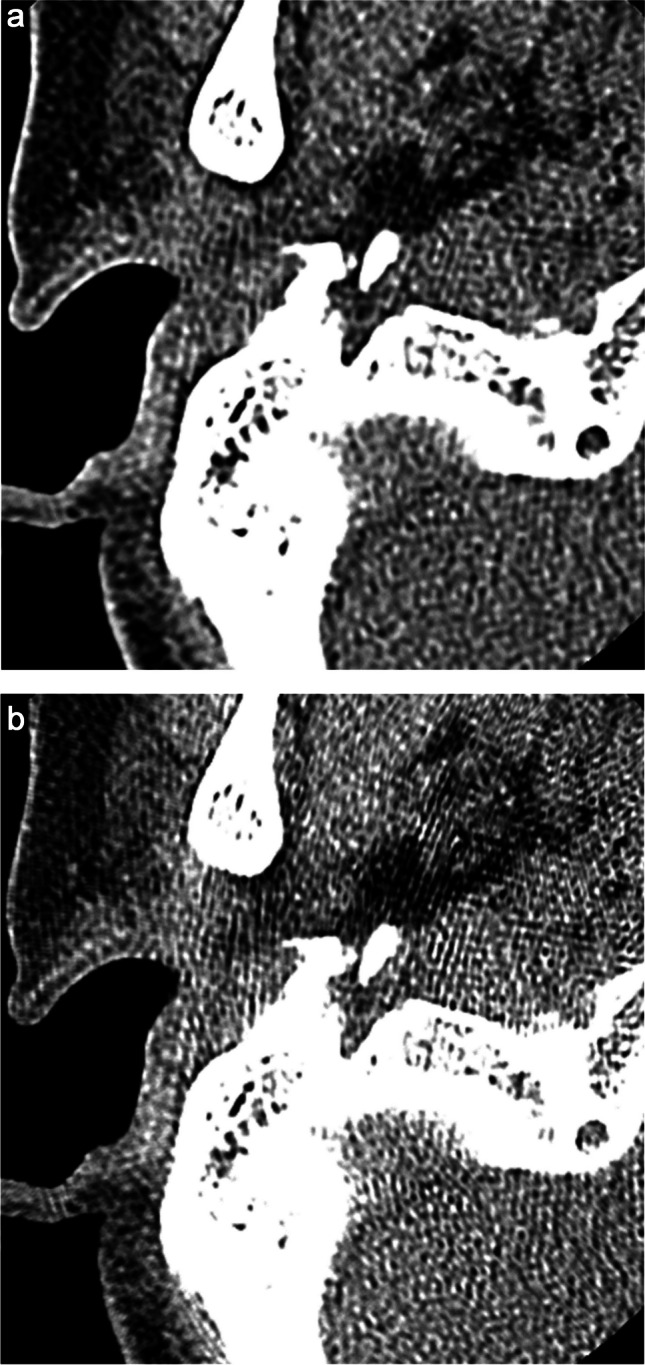
Computed tomography (CT) images of a 62-year-old female patient after **a** deep learning reconstruction (DLR) and **b** hybrid iterative reconstruction (HIR), which are displayed in the soft tissue window setting. The image noise was rated as 5/3 and 5/4 (DLR/HIR) by readers 1 and 2, respectively

Table 1 presents the results of the qualitative image analyses. Figures 2, 3, 4, and 5 contain representative CT images. The scores assigned by both readers to the depictions of the otic capsule, auditory ossicles, and tympanic membrane were statistically significantly improved for the DLR images compared with the HIR images (*p* ≤ 0.003). One reader scored the depictions of the osseous spiral lamina and cochlear axis as significantly superior for the DLR images compared with the HIR images (*p* < 0.001). Qualitative image noise was rated to be significantly reduced in the DLR images compared with the HIR images (both readers, *p* < 0.001). In addition, both readers rated the overall image quality as significantly improved in the DLR images compared with the HIR images (*p* < 0.001).

**Table 1:** Results of the qualitative image analyses

	Reader	DLR	HIR	*p* value
Depiction of				
Osseous spiral lamina	1	25/7/4/0/0	24/10/2/0/0	0.829
	2	12/19/5/0/0	0/6/30/0/0	< 0.001*
Cochlear axis	1	0/5/31/0/0	0/1/35/0/0	0.129
	2	5/24/7/0/0	0/2/34/0/0	< 0.001*
Otic capsule	1	27/8/1/0/0	7/11/18/0/0	< 0.001*
	2	12/18/6/0/0	0/1/35/0/0	< 0.001*
Auditory ossicles	1	23/7/6/0/0	15/4/17/0/0	0.001*
	2	3/19/14/0/0	0/1/35/0/0	< 0.001*
Tympanic membrane	1	10/10/16/0/0	3/5/28/0/0	0.003*
	2	5/14/17/0/0	0/0/36/0/0	< 0.001*
Noise	1	29/7/0/0/0	14/18/4/0/0	< 0.001*
	2	30/6/0/0/0	0/7/29/0/0	< 0.001*
Overall quality	1	24/12/0/0/0	10/19/7/0/0	< 0.001*
	2	12/23/1/0/0	0/1/35/0/0	< 0.001*

The numbers of patients for each score are shown. Comparisons between the reconstruction types used the Wilcoxon signed-rank test

^*^Statistically significant values (*p* < 0.050)

### Quantitative image analyses

Table 2 summarizes the data from the quantitative image analyses. CT attenuation of bone was significantly higher in the DLR images (1993 HU and 2247 HU for axial and coronal images, respectively) than in the HIR images (1769 HU and 1876 HU for axial and coronal images, respectively), *p* < 0.001 for both the axial and coronal images. On the other hand, CT attenuation of air was significantly lower in the DLR images (− 939 HU and − 955 HU for axial and coronal images, respectively) than in the HIR images (− 900 HU and − 913 HU for axial and coronal images respectively), *p* < 0.001 for both the axial and coronal images. Quantitative image noise was significantly lower in the DLR images (32.2 HU and 26.4 HU for axial and coronal images, respectively) than in the HIR images (39.4 HU and 29.0 HU for axial and coronal images respectively), *p* ≤ 0.016 for both the axial and coronal images. The CNR was significantly higher in the DLR images (98.1 and 136.0 for axial and coronal images, respectively) than in the HIR images (71.8 and 109.7 for axial and coronal images, respectively), *p* < 0.001 for both the axial and coronal images. Inter-rater concordance for CT attenuation of the bone, CT attenuation of the air, and quantitative image noise were 0.762, 0.529, and 0.311, respectively.

**Table 2:** Results of the quantitative image analyses

			DLR	HIR	*p* value
CT attenuation	Axial	Bone	1992.7 ± 56.2	1768.9 ± 47.9	< 0.001*
		Air	− 939.3 ± 27.8	− 900.1 ± 27.0	< 0.001*
	Coronal	Bone	2247.0 ± 66.1	1875.8 ± 56.0	< 0.001*
		Air	− 954.8 ± 25.1	− 913.1 ± 25.6	< 0.001*
Quantitative image noise	Axial	Air	32.2 ± 7.0	39.4 ± 6.5	< 0.001*
	Coronal	Air	26.4 ± 5.7	29.0 ± 5.2	0.016*
Contrast-to-noise ratio	Axial		98.1 ± 22.0	71.8 ± 13.3	< 0.001*
	Coronal		136.0 ± 39.6	109.7 ± 29.1	< 0.001*

Comparisons between the reconstruction types used the paired *t*-test

^*^Statistically significant values (*p* < 0.050)

The edge rise slope in DLR (2571.5 ± 923.1 HU/mm^−1^) was significantly better compared to that in HIR (2416.0 ± 846.0 HU/mm^−1^) (*p* = 0.004).

## Discussion

High-resolution CT of the temporal bone has been associated with relatively prominent image noise. In the present study, we found that DLR successfully reduced image noise and improved the depiction of the otic capsule, auditory ossicles, and tympanic membrane. Both readers in this study also rated the overall quality as significantly improved in the DLR images compared with the HIR images.

CT images of the otic capsule, auditory ossicles, and tympanic membrane can assist in diagnosing diseases such as otosclerosis, cholesteatoma, malformation, and trauma. In the present study, it became evident that depiction of those structures can be significantly improved using DLR compared with HIR. One reason for the improved depiction of temporal bone structures with DLR is likely related to reduced image noise. Several studies of DLR reconstruction in other body regions have found that image noise is significantly reduced in DLR images compared with HIR images [9–11, 19–25]. Our study, which assessed image noise in the temporal bone region, accords with those earlier studies. Another possible reason for the superior depiction of the structures of interest, especially the otic capsule and auditory ossicles, might be the higher CNR values obtained with DLR, which were based on measurements for bone and air. Higaki et al., who used a phantom study to evaluate spatial resolution with a modulation transfer function, reported a spatial frequency in task contrast of 100 HU and 200 HU with DLR, which was higher than that for HIR [31]. The potential for DLR to increase spatial resolution might be another factor in the foregoing findings. Higher edge rise slope value in DLR as compared to HIR, which was found in our study, would also support this.

In the present study, it became evident that CT attenuation of bone for the otic capsule increased in DLR images compared with HIR images. On the other hand, CT attenuation of air for the tympanic cavity decreased in DLR images compared with HIR images. Some controversy surrounds the impact of DLR on the CT attenuation of the structures. In a systematic review of abdominal CT with DLR, CT attenuation was reported to be similar with DLR and HIR [19]. However, according to Yamakuni et al., CT attenuation of the cerebral sinus was observed to be significantly different with DLR and HIR [21]. Our study results accord with the findings in the latter report. The larger difference in CT attenuation between bone and air, as well as decreased image noise, contributes to a significantly higher CNR with DLR than with HIR, as observed in our quantitative image analyses.

From a technical viewpoint, spatial resolution of CT images is negatively correlated with image noise. Thinner slices are known to be associated with increased image noise [32]. The use of a sharp reconstruction kernel is associated with higher spatial resolution and increased image noise [33]. Because high-resolution CT of the temporal bone aims to depict small structures, thinner slices and a sharper kernel are typically used, resulting in increased image noise. The DLR had been trained separately for several body regions to provide appropriate images based on clinical indications. It can be said that DLR developed for the evaluation of the inner ear balances both spatial resolution and image noise.

Our study has some limitations. First, the DLR algorithm details vary with the CT vendor. The results of our study would therefore not necessarily be applicable to DLR images obtained from other vendors’ software. Second, our study was retrospective, and patients with various conditions were included. However, significant improvements were evident in the depictions of middle ear structures. Future prospective studies to evaluate different reconstruction algorithms in specific temporal bone diseases would be warranted. Third, the inter-rater concordance for mean and standard deviation of CT attenuation in the air was not so high. However, we made efforts to enhance the reliability of data by averaging the measurements between two radiologists. Finally, although the image readers were blinded to the reconstruction algorithm used for the various images, a risk of observer bias potentially caused by the different appearance of the images produced by the various reconstruction algorithms remains.

## Conclusion

In conclusion, DLR significantly reduced image noise in high-resolution CT images of temporal bone, which resulted in improved depiction of several structures and greater overall image quality.
